# Complete mitochondrial genome of *Favonigobius gymnauchen* (BLEEKER, 1850) assembled from next-generation sequencing data

**DOI:** 10.1080/23802359.2019.1660597

**Published:** 2019-09-09

**Authors:** Hyunbin Jo, Kiyun Park, Dong-Kyun Kim, Ihn-Sil Kwak

**Affiliations:** a Fisheries Science Institute, Chonnam National University, Yeosu, Republic of Korea;; b Faculty of Marine Technology, Chonnam National University, Yeosu 59626, Republic of Korea

**Keywords:** Complete mitochondrial genome, *Favonigobius gymnauchen*

## Abstract

The complete mitochondrial genome of *Favonigobius gymnauchen* was sequenced. The circular mitochondrial genome is 16,480 bp and consists of 13 protein-coding, 2 ribosomal RNAs, and 13 transfer RNA genes (GenBank accession no. MN194589). Results of maximum likelihood analysis showed that this species clustered with other species of the family Gobbidae. This study will contribute to the phylogenetics of genus *Favonigobius* and the other genera of Gobiidae.

*Favonigobius gymnauchen* (Perciformes: Gobiidae) is a demersal fish which is important to commercial fisheries (Yamamoto and Tominaga 2005). A molecular barcoding approach in the mitochondrial region has recently been introduced to discriminate between Gobiidae species (Kim, Kweon, Kim, Kim, et al. 2004; Kim, Kweon, Kim, Lee, et al. 2004; Huang et al. 2019). Phylogenetic relationships in the family Gobiidae, however, are still poorly understood except for the cytochrome c oxidase subunit I (COI) gene (Huang et al. 2019). Furthermore, complete sequences of mitochondrial genomes (mitochondrial DNA [mtDNA]) of *F. gymnauchen* were not determined. In this study, we described a first complete mtDNA of *F. gymnauchen*, assembled using next-generation sequencing. This result will be valuable for further phylogenetic analyses of Gobiidae.

The *F. gymnauchen* specimen was collected using fishing dredge (length 1.5 m, width 0.4 m, mesh size 0.7 × 0.7 cm) from the mouth of Seomjin River, South Korea (34°59′11″N, 127°46′38″E) on 22 March 2019. The collected specimen was preserved in 99% ethanol and stored at room temperature before analysis. Total DNA was extracted from the *F. gymnauchen* sample and was stored at Specimen Museum of Fisheries Science Institute, Chonnam National University (its accession number is CNUFSI-030119001). The library preparation and DNA sequencing (100 bp mate pairs with different insert sizes, Illumina HiSeq4000) were performed at Macrogen Inc., (Seoul, Korea). The genome was assembled using the MEGA-X software (Kumar et al. 2018). The phylogenetic tree was analyzed using 13 protein-coding gene sequences using maximum likelihood (Kimura 1980). The annotated mitochondrial genome sequence of *F. gymnauchen* is available at the National Center for Biotechnology Information (NCBI) database (GenBank accession number MN194589).

The circular mtDNA of *F. gymnauchen* is 16,480 bp and contains 2 rRNAs, 22 tRNAs, and 13 protein-coding genes. The A + T base composition of the genome is 51.92%, the A + T content of genes ranges from 38.89 to 65.71%. The most common start codon was ATG. However, two genes (cox1 and nad6) were TAC and TAC. Seven genes were terminated with a complete TAA stop codon and the rest were incomplete.

Phylogenetic analysis based on the mitochondrial genomes of 13 species by MEGA-X showed that *F. gymnauchen* clustered with the clade of *Mugilogobius abei* supported by a low bootstrap value (44%) and the family Gobiidae was not a monophyly (Figure 1), in which the similar mtDNA sequences of the order Perciformes were downloaded from GenBank through BLASTN and crucian carp (*Carassius auratus*) of the order Cypriniformes was used as outgroup. Considering that the genome of the family of *F. gymnauchen* has been relatively poorly understood, the elucidation of the complete mitochondrial genome sequence of this species will be extremely useful for future Gobiidae phylogeographical studies.

**Figure 1.**
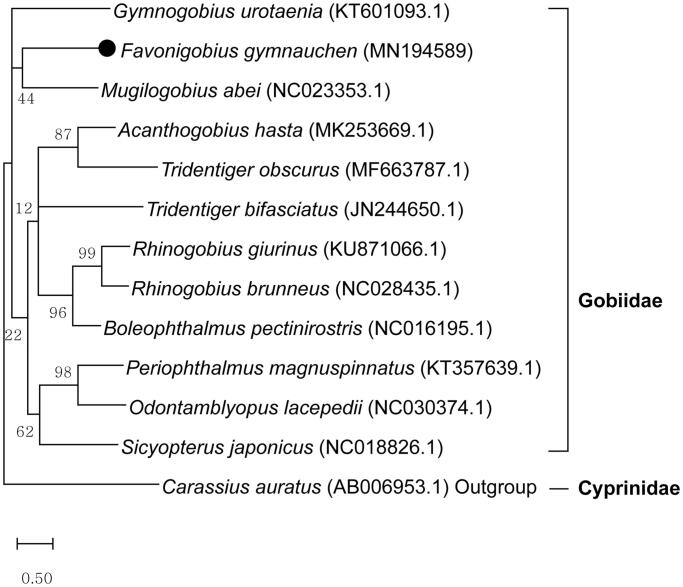
Maximum likelihood phylogenetic tree based on mitochondrial genome sequences. All the bootstrap values after 1000 iteration are indicated at the nodes.
